# Low Utility and Yield of Routine Outpatient CT Imaging in Patients With Mild Traumatic Brain Injury and Intracranial Hemorrhage

**DOI:** 10.7759/cureus.90069

**Published:** 2025-08-14

**Authors:** Connor Berger, Felipe Monteiro, Evan M Krueger, Joacir G Cordeiro, Ronald Benveniste

**Affiliations:** 1 Neurosurgery, University of Miami, Miami, USA; 2 Neurosurgery, Advocate Health Care, Downers Grove, USA; 3 Neurosurgery, University of Miami Miller School of Medicine, Jackson Memorial Hospital, Miami, USA

**Keywords:** computed tomography, intracranial hemorrhage, mild traumatic brain injury, outpatient follow up, resource utilization

## Abstract

Objective and background: This study aims to better characterize the utility of CT scan imaging when seeing mild traumatic brain injury (TBI) patients managed non-operatively in a clinic. The benefit of routinely scheduling outpatient head CT for patients discharged with mild TBI and intracranial hemorrhage (ICH) remains unclear. Unselective imaging increases cost, scanner demand, and cumulative radiation exposure.

Methods: We performed a retrospective cohort study of 100 consecutive adults with mild TBI (Glasgow coma scale (GCS) 13-15) and non‑operative ICH who were admitted between January 2021 and December 2022, returned to our neurosurgery clinic one to four weeks after discharge, and underwent protocol‑driven follow‑up CT. Demographics, injury characteristics, inpatient course, clinic findings, and CT results were abstracted. Radiographic progression (new hemorrhage or ≥25% volume increase) was the primary outcome. Univariate tests and stepwise multivariable logistic regression explored predictors (p < 0.05).

Results: The mean age was 53.8 ± 20.2 years; 63/100 (63 %) were men. Subdural hematoma occurred in 38/100 (38%), contusion in 20/100 (20%), and epidural hematoma in 7/100 (7%). At the clinic review, 37/100 (37%) reported persistent or new symptoms, and 4/100 (4%) had a new focal neurological deficit. Follow‑up CT demonstrated radiographic progression in 4/100 (4%); only 1/100 (1%) required surgical evacuation of a chronic subdural hematoma. Anticoagulation 6/100 (6%), antiplatelet therapy 18/100 (18%), hemorrhage subtype, and inpatient enlargement were not associated with delayed progression on univariate (all p > 0.20) or multivariable analysis (area under the receiver operating characteristic curve (AUROC) 0.58).

Conclusion: In clinically stable mild‑TBI patients with ICH, routine outpatient CT changed management in only 1% of cases. A symptom- or risk‑based imaging strategy appears safe and could markedly reduce radiation exposure, scanner congestion, and cost.

## Introduction

Mild traumatic brain injury (TBI), defined by a Glasgow coma scale (GCS) score of 13-15, represents roughly 90% of all head‑injury presentations and carries an estimated annual incidence of 0.1%-0.3% in the general population [1]. The CT scans identify acute intracranial hemorrhage (ICH) in about 8% of these patients [2] and in 9% of those receiving oral anticoagulation at the time of injury [3]. Once ICH is confirmed, clinicians must decide whether and when additional imaging is warranted.

Follow‑up CT can (i) detect hematoma enlargement before neurological decline, (ii) inform the need for continued admission versus discharge, and (iii) guide safe resumption of antithrombotic therapy. Counterbalancing these benefits, each scan costs a median US$1,900 and delivers approximately 2 mSv of ionizing radiation, conferring a lifetime fatal‑cancer risk of ≈ 1 in 10,000 [4]. Nationwide utilization exceeds 70 million CT examinations annually, and modeling studies project > 20,000 future cancers attributable to current imaging patterns [5].

Current recommendations, such as the Brain Injury Guidelines and the American College of Radiology Appropriateness Criteria®, advocate inpatient repeat CT for moderate/severe TBI or when neurological status, intoxication, sedation, anticoagulation, or a large (> 10 mL) hematoma preclude reliable examination [6,7]. These guidelines offer little direction for outpatient imaging in clinically stable mild‑TBI patients once they have left the hospital.

Evidence addressing this question remains limited. Three single-center retrospective series have reported radiographic progression rates of 0% to 4% and surgical intervention rates of 0% to 1% when routine CT scans were obtained one to six weeks post-discharge [8-11]. Parallel work is exploring serum biomarkers (e.g., glial fibrillary acidic protein and ubiquitin‑C‑terminal hydrolase‑L1) and advanced neuroimaging to improve risk stratification, but these tools are not yet validated for routine outpatient use [12]. A meta‑analysis of 39 cohorts (n = 23,475) calculated a pooled 2.5 % risk of deterioration among GCS 13‑15 patients with ICH, with most events occurring within 48 hours, well before typical clinic review [13]. Heterogeneity in inclusion criteria, hemorrhage subtype, antithrombotic exposure, and imaging intervals limits generalizability.

At our center, all mild‑TBI patients with ICH have historically been scheduled for routine pre‑clinic CT. We therefore undertook a retrospective cohort study to quantify delayed radiographic progression, downstream testing, and clinical impact of this practice, and to explore potential predictors of late progression in an unselected, contemporary population.

## Materials and methods

Study design and setting

The objective was to observe how head CTs before pre-clinic examination show low utility in the post-injury outpatient setting. This is a single‑center retrospective cohort study conducted at the University of Miami Hospital, a level‑I American College of Surgeons‑verified trauma center in Miami, Florida, USA. The study window spanned 1 January 2021 to 31 December 2022. A total of 148 adult discharges with mild‑TBI ICH were screened during the study window; 48 (32%) lacked in‑system follow‑up imaging and were excluded, leaving 100/148 (68%) patients for analysis. The protocol was reviewed and approved by the University of Miami Institutional Review Board (approval no. 20201030). The requirement for written informed consent was waived owing to the retrospective nature of the investigation. The study adheres to the Strengthening the Reporting of Observational Studies in Epidemiology (STROBE) statement for cohort research.

Sampling technique

A consecutive sampling strategy was employed. The electronic medical record (Epic Systems; Epic Systems Corp., Verona, WI, USA) was queried for adult (≥18 years) discharges with ICD‑10 codes S06.5‑S06.9 (intracranial injury) whose initial head‑CT report contained the keywords “subdural,” “epidural,” “contusion,” or “subarachnoid.” The query output was cross‑referenced with the trauma registry to ensure capture completeness. A formal a priori calculation was not undertaken. The cohort size reflected the total number of consecutive cases meeting inclusion criteria in the 24‑month window, consistent with prior observational work.

Eligibility criteria

Inclusion criteria comprised mild TBI with an admission GCS of 13-15, acute intracranial hemorrhage confirmed on the index head CT, no neurosurgical intervention during the index admission, and a routine outpatient head CT obtained within 30 days of discharge, together with a documented neurosurgical clinic examination. The exclusion criteria were age <18 years, moderate or severe TBI (GCS ≤ 12), penetrating cranial injury or polytrauma necessitating intensive‑care admission longer than 48 hours, pregnancy, follow‑up imaging available only at outside institutions or missing from the archive, and death before the scheduled outpatient visit. No deaths occurred during the study period.

Data collection and variable definitions

Data was extracted into a standardized REDCap (Vanderbilt University, Nashville, TN, USA) instrument capturing demographics (age and sex); mechanism of injury (motor‑vehicle collision, fall, pedestrian‑struck, assault, or other); intoxication status (blood‑alcohol level > 0.08 g/dL or documented clinical intoxication); anticoagulant or antiplatelet therapy at presentation; admission clinical findings (GCS, focal neurological deficit, seizure); index CT variables (hemorrhage subtype, hematoma volume calculated using the ABC/2 method for subdural hematoma (SDH) or epidural hematoma (EDH), presence of local mass‑effect, midline shift); in‑patient radiographic enlargement (≥ 25 % volume increase or any new hemorrhage on repeat CT); and outpatient parameters, including days to clinic, new or persistent symptoms, new focal deficit, follow‑up CT findings, additional imaging, and surgery or readmission within 90 days.

Imaging review and outcome measures

All follow‑up CT scans were independently reviewed by a fellowship‑trained neuroradiologist and a board‑certified neurosurgeon blinded to the initial reports. Interobserver reliability for progression was κ = 0.81; discrepancies were resolved by consensus. The primary outcome assessed was radiographic progression (new hemorrhage or ≥25% volume increase). Secondary outcomes included the need for additional imaging, neurosurgical intervention, or hospital readmission.

Statistical analysis

Analyses were performed using SPSS Statistics version 29.0 (IBM Corp., Armonk, NY, USA). Continuous variables were assessed for normality with Shapiro‑Wilk and reported as mean ± SD or median [IQR]; categorical data as n (%). Student’s t‑test or Mann‑Whitney U compared continuous variables; χ² or Fisher’s exact compared categorical variables. Variables with p < 0.20 on univariate testing (age, sex, intoxication, anticoagulation, antiplatelet use, and inpatient enlargement) were entered into a stepwise multivariable logistic regression model to identify predictors of outpatient progression. Model discrimination was evaluated with the area‑under‑the‑receiver‑operating‑curve (AUROC). Two‑tailed p-value < 0.05 defined statistical significance. Missing data were < 5% for all variables and managed with complete‑case analysis.

## Results

Statistical analysis

A chi-square analysis was conducted to compare the association between anticoagulant use and hemorrhagic progression on follow-up CT. No statistically significant association was found (χ² = 0.82, p = 0.37), suggesting that anticoagulant use alone is not a reliable predictor of worsening hemorrhage. Furthermore, logistic regression analysis demonstrated that none of the evaluated variables (age, initial hemorrhage type, and presence of symptoms at follow-up) were statistically significant predictors of hemorrhagic progression (p > 0.05 for all variables). The area under the curve (AUC) for this model was 0.58, reflecting poor discrimination. A total of 148 adult discharges with mild‑TBI ICH were screened during the study window; 48 (32%) lacked in‑system follow‑up imaging and were excluded, leaving 100/148 (68%) patients for analysis. 

Patient characteristics

Key demographic and admission characteristics are summarized in Table 1. Mean age was 53.8 ± 20.2 years; 63/100 (63%) were male. Motor vehicle collision was the leading mechanism (49/100, 49%), followed by falls (35/100, 35%). Twenty‑two patients (22%) were intoxicated on arrival. Anticoagulant therapy was documented in 6/100 (6%), and antiplatelet therapy in 18/100 (18%). Admission GCS was 15 in 60/100 (60%), 14 in 29/100 (29%), and 13 in 11/100 (11%); focal neurological deficit was present in 3/100 (3%).

**Table 1:** Demographics and admission data (total n = 100) GCS: Glasgow coma scale

Variable	Category	n (%)	Test statistic
Age, years (mean ± SD)	—	53.8 ± 20.2	—
Sex	Male	63	—
	Female	37	—
Intoxication on arrival	Yes	22	χ² = 0.72
	No	78	—
Anticoagulant use	Yes	6	χ² = 0.82
	No	94	—
Antiplatelet use	Yes	18	χ² = 0.10
	No	82	—
Admission GCS	15	60	—
	14	29	—
	13	11	—
Mechanism of injury	Motor vehicle collision	49	—
	Fall	35	—
	Pedestrian‑struck	8	—
	Assault	5	—
	Other	3	—

In-hospital imaging findings

Imaging findings during hospitalization are shown in Table 2. Subdural hematoma (38%) and contusions (20%) were the most frequent hemorrhage types, while epidural hematoma was seen in 7%. Approximately 21% of patients had multifocal hemorrhages (e.g., subdural plus subarachnoid). Local mass effect was noted in 14%, and 9% had midline shift, although none required surgical evacuation. Worsening hemorrhage on inpatient follow-up CT was observed in 24% of patients, but again, no inpatient surgeries were performed.

**Table 2:** Index CT characteristics (total n = 100) Note that values are patient counts (%) for each CT feature.

Finding	n (%)
Subdural hematoma	38
Contusion	20
Subarachnoid hemorrhage	14
Epidural hematoma	7
Multiple compartments	21
Local mass‑effect	14
Midline shift > 5 mm	9
In‑patient enlargement	24

Outpatient clinic review

The median interval to follow-up was 20 days (IQR 15-26). Persistent or new symptoms were reported by 37/100 (37%), namely headache (14), dizziness (eight), cognitive complaints (1), and ≥ 2 symptoms (14). A new focal neurological deficit was documented in 4/100 (4%). The follow‑up CT demonstrated radiographic progression in 4/100 (4%): two enlarging subdurals, one new hygroma, and one new trace subarachnoid hemorrhage (SAH). Additional imaging (MRI or repeat CT) was ordered for 7/100 (7%) patients. Ultimately, 1/100 (1%) underwent surgical evacuation/embolization of a chronic subdural hematoma. Detailed cases are presented in Table 3.

**Table 3:** Patients Requiring Additional Imaging or Surgery ICH: Intracerebral hemorrhage, SDH: Subdural hematoma, EDH: Epidural hematoma, OSH: Outside hospital, MMA: Middle meningeal artery embolization

Case	Age/Sex	Initial ICH type	Clinical symptoms	Follow‑up CT	Antithrombotic	Outcome
1	53 M	SDH	Persistent dizziness	Larger SDH (ER 3 months)	None	Non‑operative
2	66 F	SDH + Contusion	Headache	Smaller SDH	None	Symptoms resolved
3	61 M	EDH	Headache ± hearing loss	Stable EDH → resolved	None	Observation
4	27 M	Contusion	Asymptomatic	MRI resolved; incidental aneurysm	None	OSH follow‑up
5	25 M	Contusion	Asymptomatic	New hygroma → enlarging chronic SDH	None	Burr holes + MMA
6	46 M	SDH	Headaches	Smaller SDH	None	Observation
7	61 M	SDH	Asymptomatic	Larger SDH	None	Lost to follow-up

Univariate analysis and multivariable logistic regression

**Figure 1:**
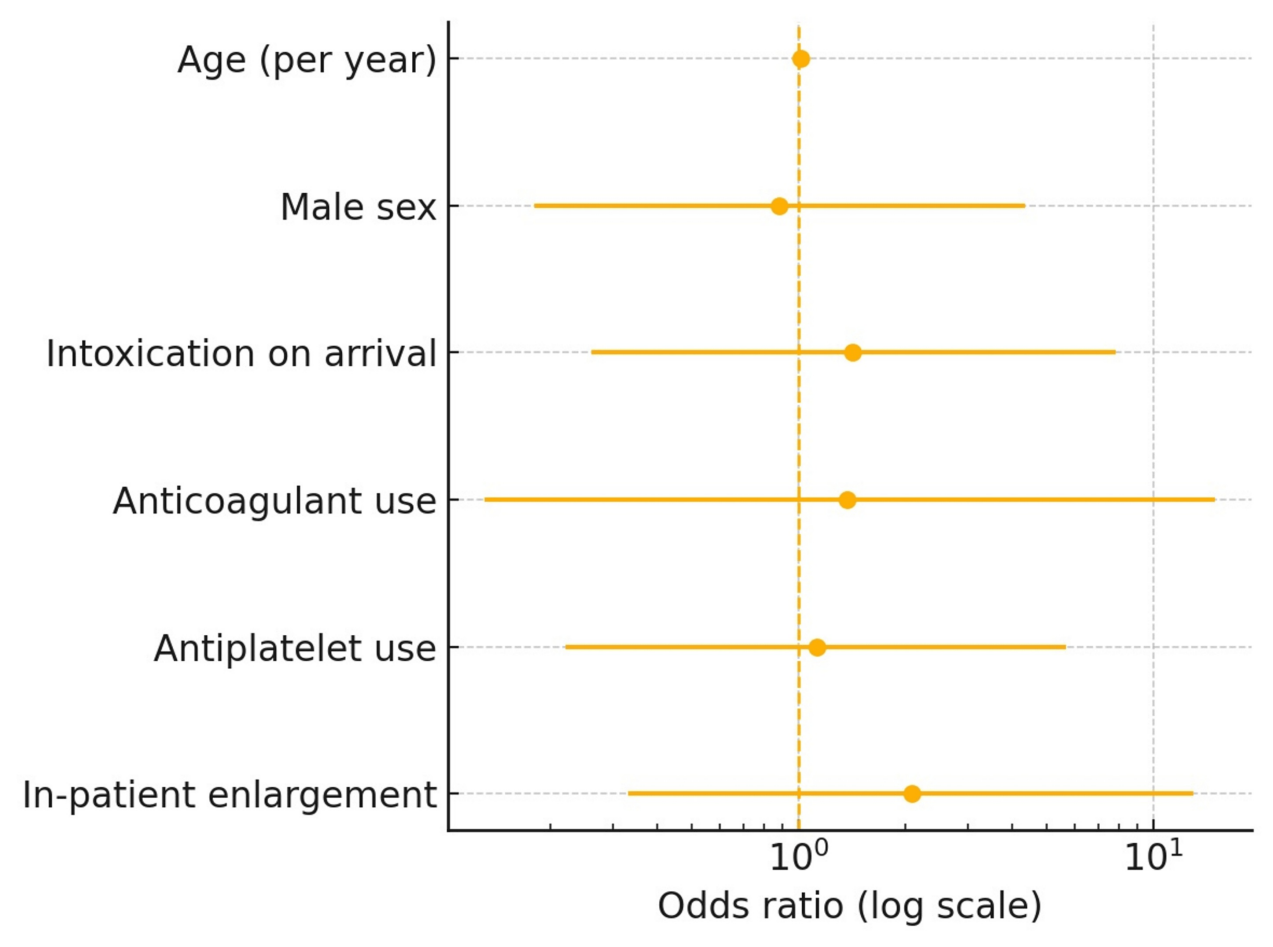
Forest plot of multivariable logistic-regression predictors of outpatient radiographic progression (total n = 100) The points are odds ratios, the horizontal bars denote 95% confidence intervals, and the vertical dashed line indicates odds ratio = 1.

In univariate testing, anticoagulation (χ² = 0.82, p = 0.37), antiplatelet therapy (χ² = 0.10, p = 0.75), and inpatient enlargement (χ² = 1.26, p = 0.26) were not associated with outpatient hemorrhage progression, and no other variable achieved statistical significance (p > 0.20). Variables with p < 0.20 in univariate screening (age, sex, intoxication, anticoagulation, antiplatelet use, and inpatient enlargement) were entered into a stepwise model for predicting outpatient progression. No predictor achieved significance; the final model yielded AUROC 0.58 (95 % CI 0.41-0.76), indicating poor discriminatory capability (Table 4, Figure 1). Radiographic progression after discharge occurred in 4/100 (4%), and only 1/100 (1%) patients required surgical intervention. Importantly, no clinical or radiological variable, including anticoagulation, antiplatelet therapy, hemorrhage subtype, or inpatient enlargement, reliably predicted delayed progression.

**Table 4:** Multivariable predictors of outpatient progression (total n = 100) Variables entered from univariate p <0.20 and outcomes.

Variable	Odds ratio	95 % CI
Age (per year)	1.01	0.97‑1.05
Male sex	0.88	0.18‑4.33
Intoxication	1.42	0.26‑7.80
Anticoagulant use	1.37	0.13‑14.9
Antiplatelet use	1.12	0.22‑5.66
Inpatient enlargement	2.08	0.33‑12.9

## Discussion

Principal findings

In this single‑center cohort of 100 patients with mild TBI and non‑operative ICH who underwent routine follow‑up CT one to four weeks post‑discharge, radiographic progression occurred in only 4/100 (4%) and resulted in surgical management in 1/100 (1%). These figures mirror the low‑yield range (0% to 4% progression; 0% to 1% surgery) reported by prior series [6‑8,11] and underscore that scheduled imaging rarely influences outpatient management when the early inpatient course is uneventful.

Comparison with existing evidence

Rubino et al. detected new or enlarging lesions in 2% (2/94) of outpatients with cerebral contusion or traumatic SAH, with no surgical interventions [8]. Befeler et al. observed radiographic change in 4% (10/226) without operations [9]. Weber et al. reported progression in 4% (6/148) and no treatment change [10]. A pooled analysis of 39 studies (n = 23 475) by Marincowitz et al. estimated a 2.5 % risk of clinical or radiographic deterioration in GCS 13-15 patients with ICH, predominantly within 48 hours of injury [13]. Our contemporary cohort, encompassing diverse hemorrhage subtypes and 24/100 (24%) on antithrombotic therapy, demonstrates similarly minimal delayed progression.

Radiation dose and economic considerations

A non-contrast head CT delivers ~2 mSv-≈8 months of background radiation with an estimated lifetime fatal‑cancer risk of 1 in 10,000 per adult scan and higher in children [4,14]. Pearce et al. demonstrated a three‑fold increase in brain‑tumor risk after two or more pediatric head CTs [14]. Though our cohort was adult, many patients accrued ≥3 scans between admission and follow‑up, highlighting cumulative exposure. At a median charge of US $1 900 per scan [5], the protocol generated ~US $190 000 in direct imaging costs for 100 patients, yet altered management in only one, a cost‑per‑actionable‑scan of ~US $190 000.

Risk stratification

Neither anticoagulation (OR 1.4, 95% CI 0.2-9.2) nor antiplatelet use (OR 1.1, 95% CI 0.2-6.2) predicted progression in multivariable analysis, aligning with meta‑analytic evidence that anticoagulation elevates initial ICH risk but minimally influences late enlargement once the inpatient period is stable [2,3]. Likewise, hemorrhage subtype, inpatient enlargement, hematoma volume, and midline shift failed to predict outpatient progression (all p > 0.05), emphasizing the challenge of developing reliable delayed‑imaging tools in a low‑event setting. Elderly patients can develop delayed chronic subdural hematoma weeks after injury [15]. Interestingly, the lone surgical case in our study was a 25‑year‑old who developed a chronic SDH; thus, age alone was not predictive in this small sample. Larger prospective datasets are needed to clarify whether advanced age, large initial hematoma (> 10 mL), cerebral atrophy, or antithrombotic therapy justify routine imaging.

Implications for practice

Our findings and the extant literature support a selective algorithm in which follow‑up CT is reserved for patients who develop new or worsening neurological findings, possess large initial hematomas with mass‑effect, or have high‑risk features for subacute expansion (e.g., uncontrolled anticoagulation). Adopting such criteria could substantially reduce radiation exposure, scanner backlog, and healthcare costs without compromising safety.

Strengths and limitations

Strengths include a clearly defined cohort, blinded dual‑reader imaging assessment (κ = 0.81), and integrated clinical follow‑up within one health system. Limitations encompass retrospective design, single‑center scope, modest sample size limiting multivariable power, and exclusion of 48/148 (32%) patients who obtained follow‑up imaging externally, potentially under‑ or over‑estimating progression. This exclusion was necessary, however, as double-blinded reviewers of imaging are needed to ensure accuracy in image analysis and consistency. Head CT has a low likelihood of progression >30 days post injury as shown in the literature, so limited follow-up is unlikely to show a significant clinical difference per Joseph et al. [16]. Follow-up imaging >2 weeks post-discharge showed no cases of delayed worsening. Most delayed worsening occurred within seven days. There was no evidence of worsening for >30 days. No deaths occurred in the study period. Future multi‑center prospective registries with standardized imaging timelines are required to refine prediction models for delayed hemorrhage progression. Integration of quantitative CT metrics (volumetrics, radiomics) and serum biomarkers such as GFAP and UCH‑L1 may improve risk stratification and merit systematic evaluation [12].

## Conclusions

Routine outpatient head CT performed one to four weeks after discharge for clinically stable mild‑TBI patients with intracranial hemorrhage demonstrated radiographic progression in 4/100 (4%) and changed management in only 1/100 (1%). Given this low yield, a selective imaging strategy appears safe for patients with new or worsening neurological findings, large initial hematomas with mass effect, or uncontrolled anticoagulation. Such a strategy could markedly reduce radiation exposure, scanner utilization, and direct costs without compromising patient outcomes.
